# Legal Regulation of Forest Therapy in Healthcare: Implications for Professional Practice in Lithuania and the Republic of Korea

**DOI:** 10.3390/healthcare14203424

**Published:** 2026-10-13

**Authors:** Gintarė Tamašauskaitė-Janickė, Daiva Petruševičienė

**Affiliations:** Department of Rehabilitation, Faculty of Nursing, Lithuanian University of Health Sciences, LT-50161 Kaunas, Lithuania; daiva.petruseviciene@lsmu.lt

**Keywords:** certification, forest bathing, nature-based interventions, legal regulation, health system, forest therapy, forest therapy specialist, professional forest therapy, professional requirements, law

## Abstract

**Highlights:**

**What are the main findings?**

• Forest therapy is governed by diverse and non-harmonized legal and professional frameworks, with significant differences in legal conception, definitions, qualifications, training, certification, and accreditation requirements.
• Regulatory differences may have consequences for professional forest therapy practice and service, professional accountability, service quality, and the protection of users’ and patients’ rights, particularly when forest therapy is provided in healthcare settings.

**What are the implications of the main findings?**

• Clearer public professional forest therapy standards and qualification requirements may be relevant to legal uncertainty and support consistent, high-quality professional forest therapy services.
• Greater regulatory clarity and coordination between forest therapy and healthcare frameworks may have implications for professional mobility, regulatory certainty, professional accountability, and protection of patients’ and service users’ rights while preserving flexibility in this emerging field.

**Abstract:**

**Background/Objectives:** This article examines legal and professional challenges related to legal regulation of forest therapy. It explores the legal and institutional frameworks governing professional forest therapy and their implications for professional qualifications, access to practice, and the organization of professional activities in Lithuania and the Republic of Korea. It also illustrates regulatory models and examples of certification practices for forest therapy professionals in the European Union (EU) and third countries, provided by public and private bodies. This study identifies similarities and differences in professional forest therapy training and qualification requirements, and considers their potential implications for professional responsibility and protecting service users. **Methods:** A structured comparative legal analysis of legal and documentary sources was conducted, complemented by a structured analysis of scientific literature. **Results:** Forest therapy is regulated through diverse, often non-harmonized legal and professional frameworks. Differences exist in the legal definition of the practice, practitioner qualifications and training, and certification and accreditation procedures. These differences may affect access to professional practice, professional accountability, and safeguards for users, especially where professional forest therapy is provided in a healthcare context. **Conclusions:** The lack of harmonized regulation may have relevance for legal uncertainty among practitioners and service users. Greater clarity on professional forest therapy standards, methodology, qualifications, and the relationship between forest therapy and healthcare regulation can be relevant to professional mobility, forest therapy service quality and consistency, and the protection of patients’ and users’ rights.

## 1. Introduction

Worldwide, interest in forest therapy, forest bathing, and other nature-based interventions and practices is increasing, as demonstrated by recent bibliometric analyses documenting the growth and diversification of research in this field and its potential applications [1,2,3]. A systematic review of the systematic reviews and meta-analyses on the psychological and physical effects of forests on human health showed that forest-based interventions have beneficial effects on cardiovascular, immunological/inflammatory, and mental health outcomes in both healthy individuals and those with pre-existing conditions, and noted the lack of consistent use of the terms forest bathing (Shinrin-Yoku), forest therapy, or forest medicine, highlighting the need for future studies to clearly define the content of their interventions [4] (p. 30). On the one hand, many scientific studies have researched the effects of forest therapy on the healthcare and public health [5,6,7,8,9,10,11] (pp. 20–37 in ref. [8]), education [12,13,14], and tourism [15,16,17,18] (pp. 95–105 in ref. [15]). These studies do not provide a detailed analysis of legal issues; however, they highlight the intersectoral connections of forest therapy as a method.

On the other hand, certain authors reveal attempts to guide forest therapy through institutionalization within the framework of forest policy implementation by discussing that legislation on forest welfare services requires further development through binding commitments at the EU and national levels, supported by a clear European vision and strengthened by national regulations and local good practices [19].

The practical integration of nature-based interventions into the socially sensitive healthcare sphere requires a deeper understanding of the legal and regulatory environment for applying such methods. To ensure patients’ right to health, healthcare is subject to stricter legal regulations; various requirements and restrictions on economic activities are established, and the conditions or limitations for such activities are specified in the context of health systems. In this context, these circumstances create preconditions to assess how legal regulation affects the professional opportunities of forest therapy specialists to provide forest therapy services in their countries of establishment and beyond their borders.

For this study, a conceptual and terminological framework is applied to distinguish between related but not necessarily synonymous concepts used in the literature, including forest therapy, forest bathing (Shinrin-Yoku), forest medicine, nature therapy, and nature-based interventions. These terms may overlap in practice and are used with varying meanings across scientific, professional, and regulatory contexts; therefore, they are not treated as interchangeable. In this study, these distinctions serve as a working conceptual framework rather than as an attempt to establish universally accepted definitions. Within the broader field of nature-based interventions (NBIs), forest therapy can be understood as a specific form of NBI and as a health-oriented or clinical application of forest bathing (Shinrin-Yoku), particularly when structured activities are used in relation to specific physical or mental health conditions [20]. The term “forest therapy” is used as the principal working concept in this study. Within this framework, “professional forest therapy” is used as a working term to refer to forest therapy delivered within a defined professional, institutional, or regulatory context, including relevant qualification, certification, licensing, or other formal requirements. Its professional and regulatory dimensions are examined separately from the broader conceptual category of NBIs. Additionally, in this study, the comparison does not assume that Lithuania and the Republic of Korea regulate an identical legal object; rather, it examines an analogous professional function as regulated within different institutional systems.

The present study is based on the same broader research project as the authors’ previous publication [21] but addresses a distinct research question and analytical objective. Unlike the previous study, which examined the legislation governing forest therapy in healthcare and considered nature-based environments as workplaces for professional forest therapy from a labour law perspective, the present study focuses on the legal and institutional regulation of professional forest therapy in Lithuania and the Republic of Korea. It examines how the profession is legally and institutionally defined and regulated in each jurisdiction, with particular attention to professional qualifications, authorization to practise, institutional requirements, and access to practice, and incorporates distinct legal, institutional, and administrative sources, including regulatory documents and administrative data on professional training and licensing from both jurisdictions. The current analysis therefore extends the previous work by providing a more detailed examination of the regulation of professional forest therapy and access to practice within healthcare. The overlap is limited to the broader research project context, while the research question, evidence base, legal analysis, and principal analytical contribution of the present study are distinct.

The aim of this study is to comparatively examine the legal and institutional regulation of professional forest therapy in Lithuania and the Republic of Korea from a healthcare perspective. The characteristics of the regulatory ecosystem of forest therapy identified through document analysis reveal the essential features of the forest therapy phenomenon within the regulatory healthcare models, including general issues such as its legal conceptualization, the interdisciplinary integration of new professions related to the field, their systemic recognition, mandatory requirements for service provision, and the potential impact of standardization of provisions on legal liability for service providers and forest therapy specialists.

To address the identified research gap, this study focuses on the legal and institutional regulation of professional forest therapy in Lithuania and the Republic of Korea. The analysis examines how professional forest therapy is legally and institutionally defined and regulated in the two jurisdictions, with particular attention to professional qualification, authorization to practice, and institutional requirements. It further considers the regulatory implications of these differences for professional access, qualification recognition, and regulatory clarity.

It is expected that the results of the pilot research project presented in this article will make a valuable contribution from the perspective of cross-disciplinary legal studies, providing new approaches to the legal and institutional frameworks governing professional forest therapy and their implications for professional qualifications, access to practice, and the organization of professional activities.

## 2. Materials and Methods

This study employs a structured comparative legal analysis of legal and documentary sources, complemented by an analysis of scientific literature, to examine the legal and institutional framework governing professional forest therapy in healthcare. The analysis focuses on two selected jurisdictions, Lithuania and the Republic of Korea. The comparison does not assume that the two jurisdictions regulate an identical legal or professional object; rather, it examines functionally analogous forms of professional forest therapy as organized and regulated within different institutional systems. For the purposes of the comparative analysis, Lithuania is examined as representing a healthcare-licensing model, whereas the Republic of Korea represents a forest-welfare model, reflecting different institutional approaches to the organization and regulation of forest therapy.

Sources were grouped into three categories: (1) legally binding sources, including laws, regulations, ministerial orders, and regulatory authority documents; (2) scientific literature, including peer-reviewed articles, reviews, and academic books, used to provide conceptual and professional context and to examine the different uses of related terminology; and (3) professional and organizational sources, including professional associations, certification bodies, training providers, and relevant public authorities. These source categories were not treated as equivalent evidence. Legally binding sources were given priority in identifying legal requirements, institutional competences, professional requirements, and regulatory mechanisms, whereas scientific and organizational sources were used primarily to contextualize terminology, professional roles, education, training, certification, and institutional practices.

Scientific literature was identified through relevant academic databases, including Academic Search Ultimate (EBSCO), ScienceDirect, MDPI, and other interdisciplinary databases. Particular attention was given to peer-reviewed publications indexed in Scopus or Web of Science and published between 2005 and 2025. Literature was selected according to its relevance to forest therapy, professionalization, healthcare-related functions, qualifications, training, certification, and institutional organization.

Legal sources were searched through EUR-Lex for the European Union context, the Lithuanian E-TAR system, and the Korea Legislation Research Institute (KLRI) legal database for the Republic of Korea. Relevant laws, regulations, ministerial orders, and other legally relevant instruments in force up to 31 December 2025 were considered. For the legal analysis, priority was given to legally binding definitions and provisions governing the legal status of forest therapy, institutional competences, professional requirements, licensing or authorization mechanisms, qualifications, and the scope of forest therapy-related professional activities.

Case-law databases were also reviewed. The Case-Law Database of the Court of Justice of the European Union was searched for the 2005–2025 period using the term “forest therapy”, while the Lithuanian LITEKO database was searched for the 2020–2025 period using the search string “miško terapij*”. No relevant case law was identified in either database using the specified search terms and periods.

Publicly available documentary information from professional organizations, certification bodies, training providers, relevant public authorities, and forest therapy-related service providers was also reviewed. These materials were considered where they provided relevant information on professional roles, education and training, certification, institutional organization, or the practical implementation of forest therapy. This review focused on materials that were current and relevant during 2025 and Q1 2026.

The scientific literature was analyzed according to predefined analytical dimensions, including the conceptualization of forest therapy, terminology, professionalization, healthcare-related functions, qualifications, training, certification, and institutional organization. The literature was used primarily to identify and contextualize concepts and professional practices rather than to establish legally binding requirements.

The legal analysis was structured around the legal status of forest therapy, relevant legal definitions, institutional competences, professional requirements, licensing or authorization mechanisms, qualifications and training, and the scope of forest therapy-related professional practice. Where terminology differed between legal and scientific sources, legally established definitions were prioritized for the assessment of legal requirements, while scientific literature was used to explain alternative or broader uses of related concepts.

The comparative analysis was conducted across the same analytical dimensions in both jurisdictions while taking into account their different legal and institutional contexts. Rather than treating Lithuania and the Republic of Korea as regulating an identical profession or legal category, the analysis examined functionally analogous forms of professional forest therapy and the institutional mechanisms through which these activities are organized, recognized, qualified, and regulated. Particular attention was given to the relationship between forest therapy and healthcare regulation in Lithuania and between forest therapy and forest-welfare policy and institutional arrangements in the Republic of Korea.

The analysis also distinguished between the formal legal status of professional forest therapy and the broader professional, educational, and organizational practices surrounding it. Accordingly, statements concerning legal rights, obligations, licensing, professional authorization, and institutional competences were based primarily on legally binding sources, while statements concerning professional concepts, training practices, certification, and organizational approaches were supported by scientific and professional sources.

The comparative analysis was based on a common analytical framework covering the legal status and definition of forest therapy, professional roles, qualification and training requirements, certification and licensing, scope of practice, and institutional requirements. This study reviews the existing scientific doctrine, provides a brief overview of national regulations on the legal understanding of forest therapy and forest therapists’ professional designation, and a structured document analysis of the integration of nature therapy-related professions into health systems. This was followed by a comparative analysis of training requirements and procedures in selected regions and jurisdictions such as Lithuania and the Republic of Korea to highlight key similarities and differences. The comparative analysis adopts a functional and institutional approach, using a common analytical framework to examine both jurisdictions across the same dimensions, including legal definition and status, professional roles, qualification and training, certification and licensing, institutional requirements, scope of practice, supervision and accountability.

This study examined twelve forest therapy-related training programs offered by seven organizations across North America, Australia, Europe, and selected countries (Lithuania, Sweden, and the Republic of Korea). The analysis drew on publicly available website information on program duration, content, target audience, and certification. In addition, the analysis considered publicly available information and practical data provided by relevant stakeholders (e.g., forest therapy training or certification providers, public authorities, and other organizational websites) operating in the field. Examples from Sweden (as a Northern European comparative case), North America, and Australia were included to situate the Lithuanian and South Korean cases within a broader geographical and international context and to illustrate comparison with diverse approaches to training programs. The identified training programs are presented as illustrative examples rather than as representative of the global forest therapy training landscape, with a distinction maintained between private certification and state-recognized professional qualification or authorization to practice.

According to data from the Continuing Professional Development and Training Programme on the Competence Platform of the State Accreditation Service for Health Care Activities under the Ministry of Health [22], one forest therapy training programme was accredited in Lithuania during the 2022–2025 period. A second forest therapy training programme was accredited in Q3 2025; however, this programme was explicitly excluded from the analysis because, at the time of data collection, it had not yet commenced and was scheduled to begin in the 2025/2026 academic year. Consequently, no programme completers had been recorded, and no graduate data were available for analysis. Therefore, only the programme for which graduate data were available during the study period was included in the analysis.

Data on the number of programme graduates were obtained from the internal administrative records of the Lithuanian University of Health Sciences (LSMU). The data were based on certificates issued and recorded upon successful completion of the programme and covered the period from the programme’s commencement through Q3 2025. Access to these records was available to one of the authors in connection with their role in the management and coordination of the training programme. The administrative data were used to determine the number of programme graduates during the study period and were not treated as independently verified population-level statistics or as representative of the entire population of forest therapy-related practitioners in Lithuania.

## 3. General Legal Framework for the Regulation of Forest Therapy and Related Professions

### 3.1. Legal Concept of Forest Therapy in Healthcare Context

A systematic analysis of research resources has shown a wide range of definitions for (therapeutic) exposure to nature, and the scientific literature on nature-based interventions frequently emphasizes the lack of standardized terminology for referring to forest therapy [8] (p. 22). Examining the legal concept of forest therapy helps clarify this phenomenon. The results identify key elements of its content and application, especially in healthcare.

Although no published legal studies have specifically addressed the legal definition of forest therapy, apart from the presented pilot interdisciplinary legal study [21], interdisciplinary research on nature-based interventions commonly treats forest therapy as synonymous with or closely related to forest bathing, nature therapy practices, or related interventions that involve an individual’s mindful presence in nature and a structured engagement with natural environments using the five senses. However, several authors highlight that forest therapy is characterized by a more explicitly therapeutic intent, developed by forest medicine from Shinrin-Yoku to forest therapy as an evidence-based approach to disease prevention, emphasizing healing and the promotion of mental and physical health through respectful immersion in forest environments [15] (p. 98). The researchers conclude that forest bathing is primarily preventive for healthy individuals. In contrast, forest therapy targets therapeutic outcomes for those with existing conditions, although the distinction between the two is often unclear in practice [4] (pp. 3, 29).

The International Handbook of Forest Therapy describes forest therapy as an evidence-based public health practice through guided forest therapy walks that combine a specific blend of complementary physical and mental exercises in suitable forest surroundings, leading to lower heart rate, blood pressure, and stress levels. At the same time, it strengthens the immune system, breathing, and overall physical and mental fitness and agility [23] (p. 387). In terms of content, the description of the method relates to the international health policy approach to traditional, complementary, and integrative medicine (TCIM).

The World Health Organization (WHO) provides a general international conceptual framework for traditional, complementary, and integrative medicine, distinguishing between these concepts and defining them as follows [24] (p. ix):

• Traditional medicine “refers to codified or non-codified systems for health care and well-being comprising practices, skills, knowledge and philosophies originating in different historical, cultural contexts, that are distinct from and pre-date biomedicine, evolving scientifically for current use from an experience-based origin. It emphasizes nature-based remedies and holistic, personalized approaches to restore balance of mind, body and environment”.
• Complementary medicine “refers to additional health care practices that are not part of a country’s mainstream medicine. Evidence-based complementary medicine has the potential to support mainstream medicine and more comprehensively support people’s health and well-being needs”.
• Integrative medicine “refers to an interdisciplinary and evidence-based approach to health and well-being by using a combination of biomedical and traditional and/or complementary medical knowledge, skills and practices”.

Therefore, TCIM differs from conventional medicine, which, according to the WHO, is understood as biomedicine, “as a branch of medical science in which biological and physiological principles are applied in clinical practice, emphasizing standardized, evidence-based treatments administered by formally trained doctors, nurses, and other licensed practitioners” [24]. In its 2013 strategic document, the WHO emphasized that traditional medicine functions either as the primary healthcare system or as a support to conventional medical practices; in certain regions, traditional or non-conventional approaches are frequently described as complementary medicine [25].

When this conceptual approach is applied consistently, it can be hypothesized that forest therapy, by its nature, could potentially be classified within the TCIM domain. Nevertheless, the European Union legal framework does not provide a legal definition or framework for forest therapy, and the current legal regulatory environment of TCIM varies across EU Member States and other countries.

Lithuania was among the first European countries to regulate the TCIM field [26] and forest therapy (as part of complementary and alternative healthcare (CAHC)) [21] through legislation. The integration of TCIM into the Lithuanian legal healthcare system began in 2012, when the Government program was approved, which envisaged the aim of legally regulating the provision of non-conventional (in this study understood in accordance with the understanding of TCIM provided by the WHO) (complementary and alternative) medical services and enhancing the safety and accessibility of recreational health services [27]. In 2014, Lithuania recognized the regulation of relations concerning non-conventional medicine practices, healthy living promotion, and traditional medicine practices as a priority national legislative initiative [28].

Following almost a decade of preparatory legislative work, the special law on CAHC of the Republic of Lithuania (CAHC Law) was adopted in 2020 [29]. Although the term “natural and folk medicine” is used in the CAHC Law (as a field of complementary and alternative healthcare whose purpose is to alleviate ailments caused by diseases), its nature corresponds to the concept of traditional medicine; therefore, the authors of this article use the generalized term “traditional medicine”. This law came into force on 1 July 2021 and regulates the conditions for engaging in CAHC in the Republic of Lithuania.

In Lithuania, according to the CAHC Law, Article 2 para. 2, the CAHC is recognized as

• “State licensed healthcare provided to citizens of the Republic of Lithuania or another Member State of the European Union or a State of the European Economic Area, other natural persons exercising the rights of movement in the Member States granted to them by the legislation of the European Union, to legal entities established in the Republic of Lithuania or to legal entities established in other Member States, other organizations or their branches, as well as branches of other foreign legal persons or other organizations established in the Republic of Lithuania”,
• “which include health recreation, natural and traditional medicine and are carried out by means of research-based medicine, biological, psychological and social tools and/or empirical knowledge”.

On 13 October 2020, the Lithuanian legal system governing the CAHC field was supplemented with the list of specific services assigned to service groups and/or subgroups of the fields of supplementary and alternative healthcare, approved by Order No. V-2199 of the Minister of Health of 6 October 2020 [30]. On 5 April 2022, following the amendment of Order No. V-703 “On the Amendment of Order No. V-2199 of the Minister of Health of the Republic of Lithuania of 6 October 2020 ‘On the Approval of the List of Specific Services Assigned to Service Groups and/or Subgroups of the Fields of CAHC and of the Description of the Procedure for Its Compilation’” [31], forest therapy was incorporated into the Lithuanian CAHC services classification.

**Figure 1:**
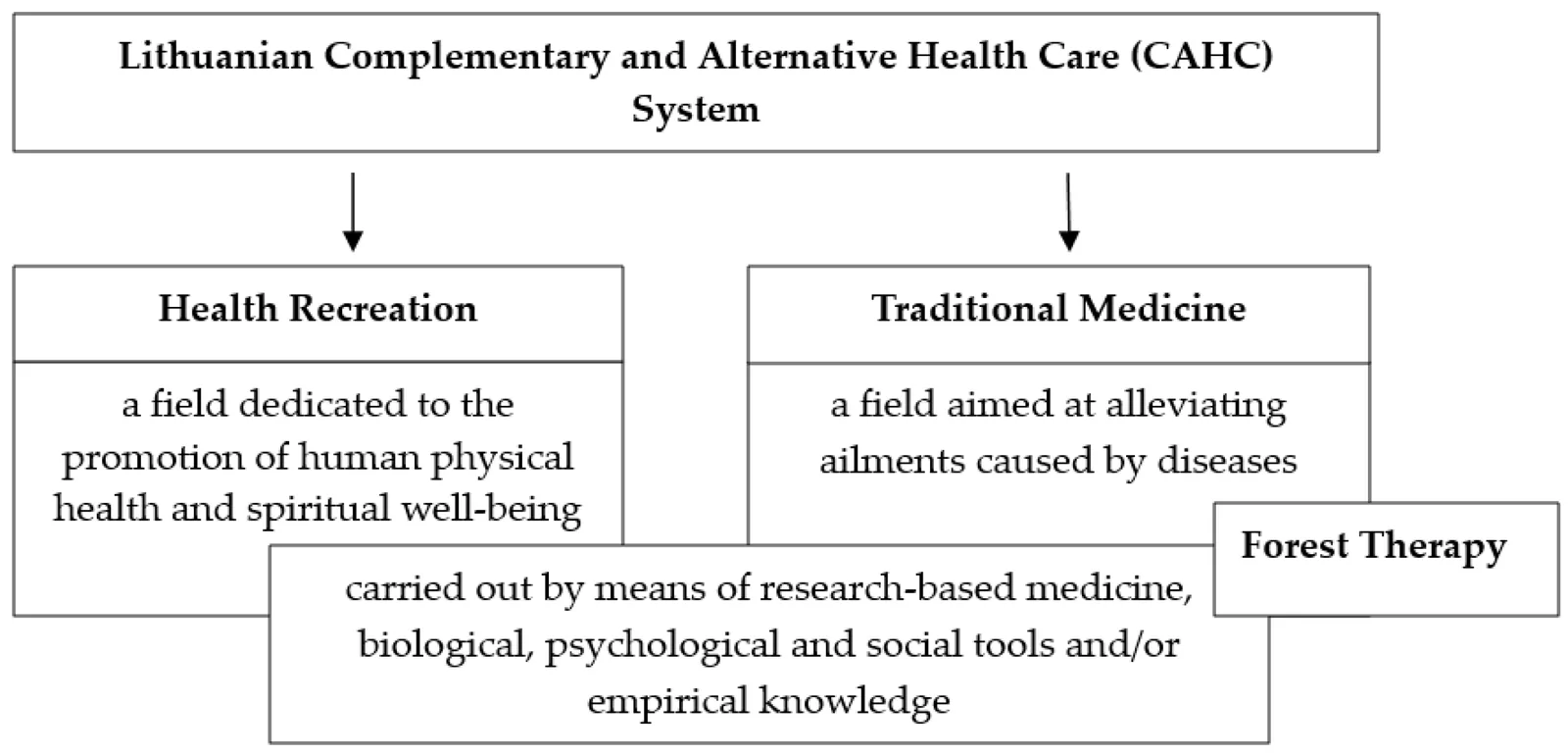
Lithuanian CAHC system in accordance with CAHC Law (data refer to 1 January 2026). Source: Compiled by the authors.

Figure 1 provides a research-based, systematic, structured legal approach to the Lithuanian CAHC system and the conceptual place of forest therapy as part of this system, according to the CAHC Law.

Finally, in 2025, the Lithuanian Medical Standard MN 189:2025 “CAHC Forest Therapy Specialist,” approved by the Ministry of Health [32] (Lithuanian CAHC Forest Therapy Specialist Medical Standard 2025), was adopted. This Lithuanian CAHC Forest Therapy Specialist Medical Standard describes the nature of forest therapy services, fields of activity, rights, duties, and competencies of forest therapy specialists. As a normative legal act, the medical standard is mandatory. It applies to all forest therapy specialists working in Lithuania, their employers, and institutions involved in training such specialists, professional development of their qualifications, and supervision of their activities.

It is also significant from legal, theoretical, and practical perspectives, as the Lithuanian CAHC Forest Therapy Specialist Medical Standard provides a legal definition of forest therapy and forest therapy service in a healthcare context.

According to the Lithuanian CAHC Forest Therapy Specialist Medical Standard, para. 3.1, forest therapy “means an effect experienced by a patient in a forest or park through the scents, sounds, sights, air, and other elements of living and non-living nature, aimed at alleviating ailments caused by diseases and health disorders, and for disease prevention and health promotion”. Pursuant to CAHC Forest Therapy Specialist Medical Standard para. 3.2, “a CAHC forest therapy service is a service provided in a CAHC institution holding a license for forest therapy within the CAHC group of biological effect services in the field of traditional medicine, by a CAHC forest therapy specialist”.

### 3.2. Emerging Professions Linked to Nature-Based Therapies

To better understand how professional work in the forest therapy field is defined in practice, the comparative study selected for analysis twelve forest therapy-related training programs offered by seven organizations providing nature-based therapy training in North America, Australia, and Europe, as well as in countries including Lithuania, Sweden, and the Republic of Korea. The data analysis was based on publicly available information from the organizations’ websites about the training courses offered, including their duration, content, target audience, and awarded certification.

Table 1 presents partial results of this study, which reveal that, in practice, there is no single established term for forest therapy specialists, with a variety of terms used to refer to specialists associated with nature-based therapy.

Regulatory issues concerning professional forest therapy specialists as a profession or professional qualification are still in their early stages. In most of the selected countries, forest therapy training courses offered online, in person, or in a hybrid format by various training providers are not subject to any legal normative requirements. Therefore, the market conditions may affect and potentially determine the price, duration, content, and form of training programs offered by training providers, and participants who complete the training receive a certificate of completion from the training organizer.

Nevertheless, in some countries, such as Lithuania and South Korea, basic issues related to professional training for specialists in nature-based therapeutic practice in the healthcare field are already regulated, and public regulatory bodies have adopted mandatory requirements for forest therapeutic training and related forest therapy services.

#### 3.2.1. Formal Professional Requirements for Forest Therapeutic Practice

##### Lithuania

Because forest therapy is recognized as a CAHC-licensed activity in Lithuania, individuals seeking to practice as CAHC forest therapy specialists must comply with formal CAHC licensing requirements.

First, the CAHC Law provides a closed and exhaustive list of professional qualifications that are mandatory prerequisites for obtaining a forest therapy specialist license. Secondly, the CAHC Law requires completion of training recognized by the Minister of Health in accordance with the established procedure.

In accordance with Article 9 Section 2 para. 3 and Article 10 Section 1 para. 2 of the CAHC Law [23], a person seeking to obtain a CAHC professional license as a forest therapy specialist must meet the following requirements:

• have a higher education degree or equivalent higher education qualification obtained after completing studies in at least one of the following fields: medicine, rehabilitation, dentistry, pharmacy, oral care, public health, nursing and midwifery, nutrition, biology, psychology, genetics, microbiology, molecular biology, biophysics, biochemistry, veterinary medicine, and
• have completed training in accordance with the procedure established by the Minister of Health.

The exception applies to the requirement to have completed training for persons who have:

• A higher education degree or an equivalent higher education qualification obtained by completing a study program in accordance with the CAHC traditional medicine biological effect services group.
• Obtained higher education in a foreign country and have had it recognized in the Republic of Lithuania in accordance with the procedure established by law.
• An equivalent higher education qualification, acquired upon completion of a study program in accordance with the CAHC traditional medicine field biological effect services group.

In Lithuania, the development of training and/or professional qualification development programs for CAHC specialists, the evaluation criteria, fields, and the procedure for coordination with and submission to the responsible institutions are regulated by the Minister of Health (Order No. V-557 2021) [40], with an amendment (Order No. V-1157 2025) in 2025 [41].

Table 2 outlines the key changes in Lithuanian CAHC forest therapy training requirements for individuals holding a professional qualification and seeking to obtain a CAHC specialist license, comparing the previous regulation applicable until 2025 with the current regulation applicable from 2026. Under the amendments, the training framework was revised from a 480 h program to a 60-ECTS program, with changes also made to the distribution and structure of theoretical and practical training components. A new limitation criterion for the temporal distribution of training is established. Because contact hours and ECTS credits measure different aspects of educational workload, the two formats are not directly quantitatively comparable.

##### The Republic of Korea

The Republic of Korea was the first country globally to establish an integrated policy for providing forest welfare services, including legal regulation, institutional management, infrastructure, and specialized expertise [19]. The authors [19] (p. 3) argue that the term Forest Welfare Services refers to social and cultural services for the population based on forests and forest resources, aimed at improving human health and well-being, and can be considered part of the Forest Cultural Ecosystem Services (FCES) concept [19] (p. 3) because it represents the means through which the benefits of FCES are delivered to a wide range of end users. Practically, these services focus on providing welfare benefits to the population through purposeful interactions with forests to enhance human health and well-being [19] (p. 3).

In South Korea, forest therapy, although primarily associated with health-related outcomes, is integrally embedded within forest policy and the regulatory framework [42]. Under this framework, the following types of Forest Welfare Services are formally recognized:

• Forest Therapy (Healing)—practices aimed at promoting human health and enhancing immunity by using natural forest elements for the prevention and treatment of diseases (e.g., forest bathing, herbal therapy, aromatherapy).
• Forest Recreation—activities supporting both passive and active relaxation, intended to restore and strengthen physical and mental health.
• Forest Education—activities designed to raise awareness and understanding of the importance of forests.
• Forest Culture—cultural events and activities taking place in forest environments.
• Forest Sports Activities—forests serving as settings and conditions for various sports and adventure activities.

The three special laws: Forestry Culture and Recreation Act 2023 [43], Forest Education Promotion Act 2021 [44], and Forest Welfare Promotion Act 2022 [45] directly regulate forest welfare services-related issues.

Table 3 presents the different categories of forest welfare experts under the legislation of the Republic of Korea.

Article 11-2 of the Forestry Culture and Recreation Act 2023 establishes the legal framework for accrediting and regulating forest healing instructors in South Korea. The Korea Forest Service is authorized to accredit qualified individuals, issue official certificates, and support their professional development in accordance with Presidential and Ministerial Decrees. Accredited forest healing instructors develop and deliver forest healing programs in designated forest environments, such as natural recreation forests, forest bathing areas, healing forests, and forest paths.

Article 11-2 of the Forestry Culture and Recreation Act 2023 also defines eligibility requirements and explicitly excludes individuals under guardianship, those with relevant criminal convictions, suspended sentences, or forfeited qualifications. Legal protection of the professional title is ensured by prohibiting its use by unqualified persons, as well as the lending or misuse of certificates and titles. Overall, under Article 11-2 of the Forestry Culture and Recreation Act 2023, forest healing instruction is formalized as a regulated professional activity, ensuring quality control, public safety, and institutional accountability within the national forest welfare system.

Thus, a forest healing instructor is a certified specialist responsible for planning, developing, and delivering tailored forest healing programs in forest welfare facilities, such as healing forests and natural recreation forests. The qualification is a Korean national professional certificate issued by the Korea Forest Service and comprises two levels [38]:

• Level 2 certification requires completion of accredited training and relevant academic education (bachelor’s degree or equivalent in health or forestry-related fields) or several years of professional experience in forest healing-related work.
• Level 1 certification is granted to experienced Level 2 instructors with at least five years of professional practice or to individuals holding advanced degrees (master’s or doctoral) or recognized engineering qualifications in relevant fields, following completion of advanced training.

#### 3.2.2. Mandatory Requirements for Forest Therapeutic Services

In Lithuania, Article 3 para. 2 of the CAHC Law specifies two essential elements of CAHC, which also apply to forest therapy as a part of the CAHC service system. Forest therapy should be provided:

• Practically—by a licensed CAHC forest therapy specialist,
• Institutionally—in a CAHC institution (legal entity) holding a license to provide forest therapy services under the category of biological effect services in the field of CAHC.

The requirements for CAHC specialists and service licensing differ depending on how services are classified into groups and subgroups.

**Figure 2:**
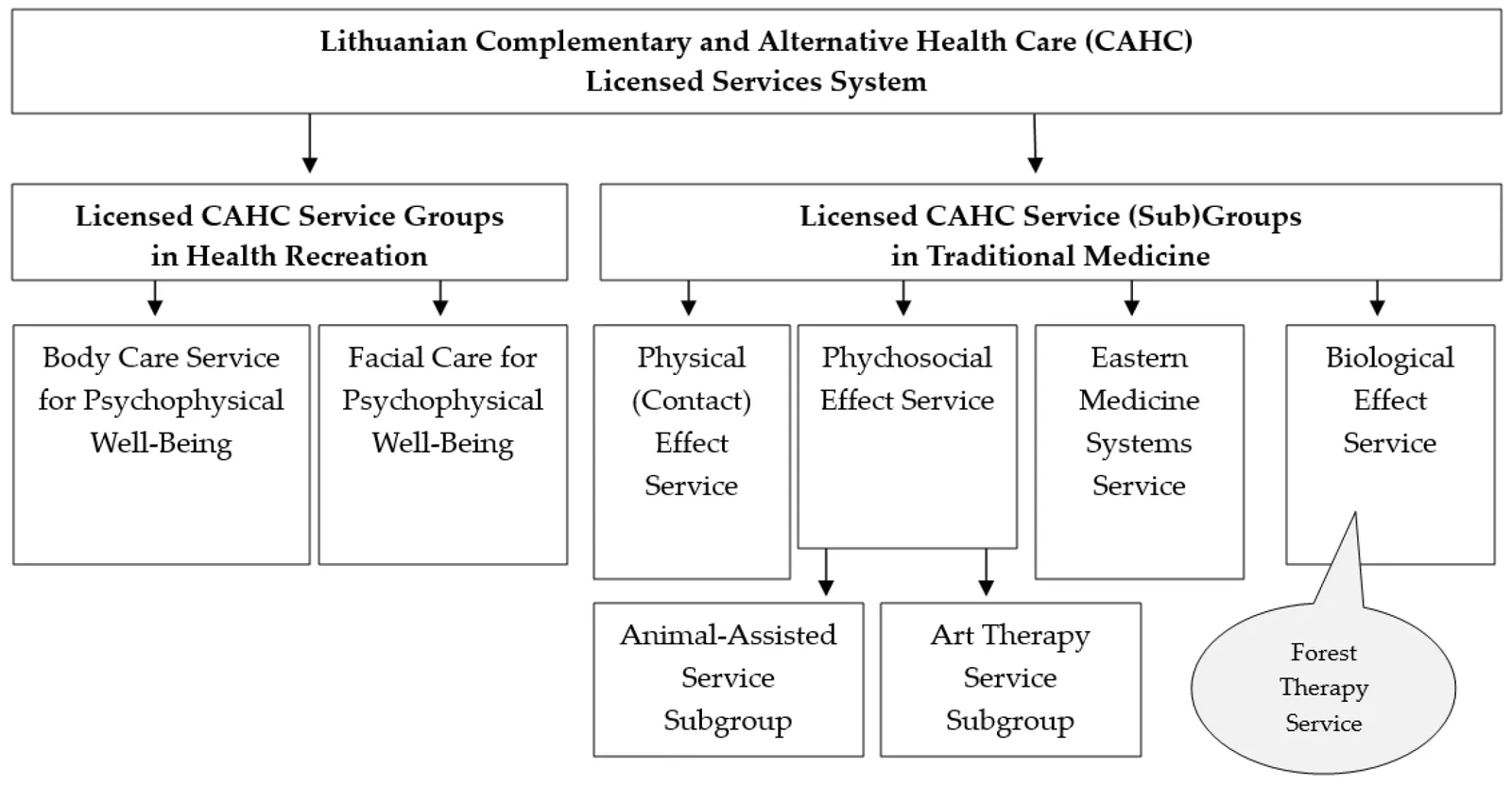
Lithuanian CAHC licensed services system in accordance with the legal CAHC framework. (Source: compiled by the authors; data refer to 1 January 2026).

Figure 2 presents a research-based, systematic, structured legal approach to the Lithuanian licensed CAHC system and the conceptual place of forest therapy within this system, according to the legal CAHC framework adopted by the Ministry of Health [30].

**Figure 3:**
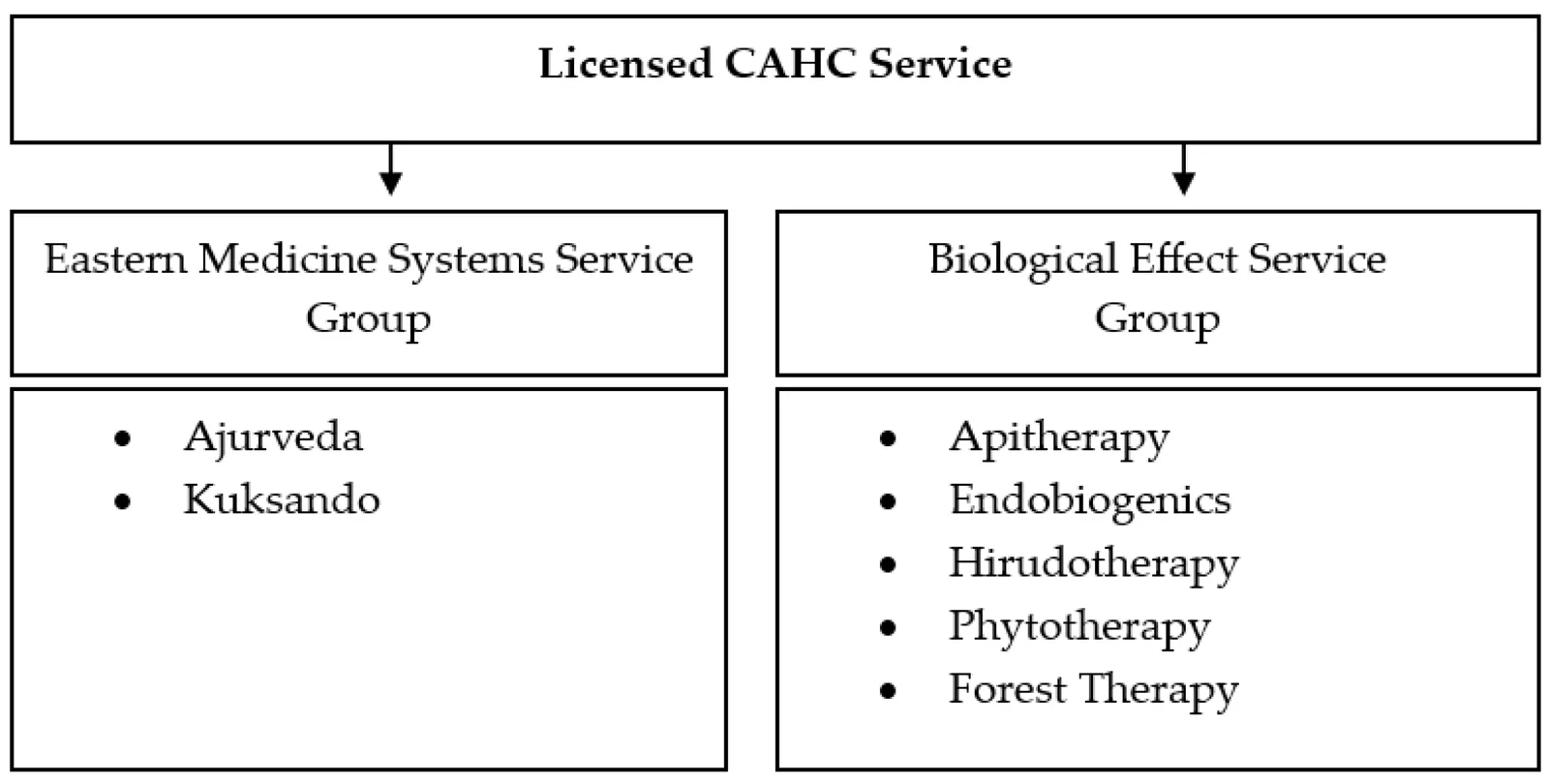
Comparative classification of Eastern medicine systems services and biological effect services within Lithuanian CAHC service groups in accordance with the legal CAHC framework (source: compiled by the authors; data refer to 1 January 2026).

Figure 3 offers a comparative allocation of Eastern medicine and biological effect services to Lithuanian CAHC service groups under the legal CAHC framework established by the Ministry of Health [24].

Thus, Figure 2 and Figure 3 show that although forest therapy originates in Japan and is derived from and, in practice, linked to forest bathing, its positive effects on physical and mental health are widely recognized in the scientific literature. Nevertheless, Lithuanian law has classified forest therapy only within the group of biological effect on health services. This group includes forest therapy, apitherapy, endobiogenics, hirudotherapy, and phytotherapy, whereas the Eastern medicine systems service group includes ajurveda and kuksando.

According to Article 5 Section 2 of the CAHC Law, legal entities seeking to provide CAHC forest therapy services must comply with the following requirements:

• Ensure that employees providing CAHC forest therapy services hold valid CAHC forest therapy specialist licenses granting the right to provide the relevant services.
• Comply with the requirements for the provision of relevant forest therapy services established in the regulations approved by the Minister of Health and/or in the CAHC service provision protocols approved by the head of the CAHC institution.
• Comply with the requirements set out in the public health safety regulations (hygiene standards) for CAHC services.
• Have civil liability insurance covering the CAHC institution. Where the insurance compensation does not fully cover the damage incurred, the CAHC institution responsible for the damage shall compensate the remaining amount equal to the difference between the actual damage and the insurance payout.

These requirements relate to protecting patients’ rights and guaranteeing compensation for potential damage resulting from the provision of CAHC services.

However, a data analysis of the practical situation regarding CAHC forest therapy service provision in Lithuania during the 2022–2025 period identified several matters for consideration.

Firstly, until 2025 Q2, the LSMU was the only pioneer institution providing forest therapy training accredited by the Ministry of Health for obtaining a forest therapy specialist license. During 2022–2025, according to data (refer to 2025 Q3) provided by LSMU, 137 participants completed the program. Of these, 38 specialists obtained forest therapy specialist licenses (official data refer to 31 December 2025) [46]. During 2023–2025, the number of institutions licensed for CAHC forest therapy service decreased from four to two by 2025 (data refer to 31 December 2025) [47].

Of the 137 individuals recorded as having completed the training program, 38 were identified as licensed professionals within the Lithuanian CAHC system, corresponding to 27.7% of the recorded graduates. This proportion describes the relationship between the number of recorded training program (accredited by the State) graduates and the number of identified licensed professionals within the CAHC framework; it should not be interpreted as the proportion of graduates who are legally permitted to provide forest therapy-related services in all professional or non-healthcare contexts. This study did not examine the reasons specialists who completed the training program chose to (not) pursue licensing procedures.

Lithuanian regulatory environment evolved during 2021–2025, particularly regarding the conditions governing professional practice and the possibility for legal entities to establish and provide forest therapy services:

• The CAHC Law established a transitional period for the application of provisions related to qualification requirements, lasting until 30 June 2025.
• Until the Medical Norm was approved in May 2025, the sector lacked regulatory clarity; therefore, potential CAHC forest therapy providers require time to adapt to changes in the regulatory environment.
• The field of nature-based therapies generally involves specialists with highly diverse professional backgrounds (primary professional qualifications). As a result, after completing forest therapy training, they may integrate part of the acquired knowledge into their ongoing activities in education, psychotherapy, counselling, tourism, or other fields on an interdisciplinary basis, which are subject to a significantly more flexible and less restrictive legal regime.

Several regulatory developments may plausibly have contributed to the relatively low proportion of legal entities obtaining licenses; however, the present study did not investigate the individual or institutional reasons underlying licensing decisions.

In summary, despite growing interest in forest therapy, the practical adaptation of forest therapy as a CAHC service in the healthcare sector to the legal CAHC regulatory framework and lawful practical activity in CAHC has been ongoing for the past five years.

#### 3.2.3. Temporary and Occasional Cross-Border Provision on Forest Therapy as CAHC Service

EU law on the free movement of workers does not provide specific provisions for forest therapy. In the Member States where CAHC forest therapy is not regulated, such activities may be carried out under general EU rules on free movement of workers or services. However, in some Member States, such as Lithuania, a special law regulates the temporary and occasional provision of CAHC services. It allows for exemptions from standard licensing requirements for CAHC forest therapy services.

According to Article 15 of the CAHC Law, EU/EEA specialists legally established in another Member State may temporarily provide CAHC services in Lithuania at the legal entity licensed for CAHC forest therapy service without a Lithuanian license for forest therapy specialist practice, subject to prior notification and submission of a declaration and supporting documents. Such providers enjoy the same rights and obligations as domestic professionals but are exempt from standard licensing, qualification reporting, and documentation requirements. For individuals established outside EU Member States, the CAHC Professionals’ Qualification Assessment Commission decides whether they may temporarily and occasionally provide CAHC services.

Differences in how forest therapy is recognized and integrated into national legal health systems affect forest therapy practitioners’ ability to engage in professional practice, as questions arise about minimum requirements for professional qualifications and competencies, as well as their recognition. However, these factors are not decisive, as legal regulation provides for exceptions that help ensure the free movement of workers.

The analysis identified provisions relevant to temporary and occasional cross-border provision of services within the Lithuanian and EU legal framework. However, the reviewed sources did not allow for a sufficiently detailed assessment of the application of the EU professional-qualification framework specifically to forest therapy as a professional activity.

### 3.3. Regulatory Models and Standardized Environment of the Forest Therapy

The conceptual framework on integration of TCIM into health systems, published by WHO in 2025 [24] (pp. 17–20), provides the general scoping review framing TCIM integration into health systems along two key dimensions:

• Decentralized vs. centralized approaches.
• Pluralistic vs. unified systems.

Combining these dimensions, four categories were identified:

• People-led—decentralized and pluralistic.
• Practitioner-led—decentralized and blended.
• Coordinated System—centralized and pluralistic.
• Blended System—centralized and unified.

The integration of TCIM models is context-dependent, with no single best approach [24]. No explicit legal provision classifying forest therapy as a component of the WHO TCIM framework was identified in the sources examined. Its potential relevance to TCIM is therefore presented as an interpretive consideration rather than an established legal classification.

The research analysis showed that, in principle, the first initiatives to standardize forest therapy at an international or regional level emerged from the interests of organizations providing commercial forest therapy training or certifying forest therapy trails and healing forests, often in collaboration with researchers, as evidenced by the three examples below.

• The International Nature and Forest Therapy Alliance (INFTA), a self-declared non-profit association founded in 2017, initiated the development of an international standard for training forest therapy guides—the International Core Curriculum for Forest Therapy (ICCFT)—with the aim of unifying diverse national approaches within a single curriculum. The ICCFT standard comprises the following key elements: the public health concept of forest therapy; the theoretical foundations of forest therapy; the planning, implementation, and evaluation of guided forest therapy walks; and a supervised guided forest therapy walk accompanied by a final written assessment [33]. The ICCFT is also linked to the first International Handbook of Forest Therapy (2019), which defines the scientific scope of this innovative, evidence-based, and timely public health approach and brings together authors from around the world to share their expertise and insights on forest therapy from diverse research perspectives [23] (p. 610).
• The International Society of Forest Therapy (ISFT) focuses on the development and standardization of forest therapy curricula, training systems, and practice, considering national and regional specificities, and seeks to make healing forests accessible to a wider audience by applying a defined catalogue of criteria that healing forests must meet as quality standards, which serves as the basis for the certification of medicinal forests [48].
• Coordinated by the Multidisciplinary Institute of Nature Therapy (MINT) at the University of British Columbia, the International Standard for Forest Therapy Practice—Framework for Health, Ecology, and Cultural Integrity [49] was published in 2025 in response to the growing diversity of forest therapy practices worldwide and the need to ensure their safe, ethical, and effective delivery. This standard is based on knowledge generated through events and initiatives in Korea, Japan, China, Sweden, Spain, and the United States. It integrates practical expertise from forest therapy training and related service providers (such as ANFT and FT Hub), as well as other field-relevant stakeholders. This standard also emphasizes the importance in the field of forest therapy of applying International Organization for Standardization (ISO) global standards setting clear benchmarks for trusted goods and services, ensuring reliability, building trust, and making choices easier for businesses and consumers: Occupational health and safety management [50], Social responsibility [51], as well as the International Union for Conservation of Nature (IUCN) Global standard for nature-based solutions [52] and WHO overview of interconnectedness [53].

At the national level, in countries where forest therapy is not regulated by normative legal acts, forest therapy training providers may adopt internal methodological standards for practice or for certifying training programs and participants.

For example, in Poland, the Relational Ecotherapy Institute publishes its Code of Ethics [54], while in Sweden, the Scandinavian Nature and Forest Therapy Institute applies an Institute’s Scandinavian/Nordic methodology and Eco Forest Therapy, expressed through three models collectively referred to as SHIFT M.A.P., which are protected as trademarks [55].

Thus, forest therapy standards are being developed at global or regional levels, most often initiated by field practitioners or related service providers such as training and certification bodies. In contrast, in some countries, certain standardizing provisions are embedded in normative legal acts regulating forest therapy issues (e.g., Lithuania and the Republic of Korea), while at the local level, individual organizations providing forest therapy develop their own small-scale standardized methodologies (e.g., Ecotherapy Poland and initiatives in Sweden). No unified standard for the practice of forest therapy in healthcare was identified within the jurisdictions and programs examined.

Nevertheless, standards adopted voluntarily have an informational and recommendatory legal effect, whereas regulatory provisions on forest therapy established in normative legal acts are binding. Consequently, the prevailing regulatory model directly shapes the regulatory and competitive environment of forest therapy. At the same time, different regulatory approaches may have varying implications for protecting patients’—as potential users of forest therapy services—legitimate interests, especially from the perspective of healthcare expectations.

## 4. Discussion

The conducted research demonstrates that the legal regulation of forest therapy and related professions within the healthcare domain remains fragmented. At the international and EU levels, no specific building legal instruments govern the professional forest therapy relationships related to professional qualifications, access to practice, and the organization of professional activities.

In countries where forest therapy is de jure integrated into the health system (e.g., Lithuania), mandatory licensing requirements apply to both forest therapy specialists and CAHC institutions providing forest therapy services. In the Republic of Korea, forest therapy is legally embedded within forest policy as a forest-based ecosystem service, with regulatory provisions focusing on the training and certification of forest therapy specialists operating within forestry and forest welfare public institutions. The professional requirements identified in the two jurisdictions reflect different institutional and legal frameworks rather than different levels of regulation of an equivalent occupation. Accordingly, the Lithuanian CAHC forest therapy specialist and the Korean forest therapy instructor are treated in this study as functionally analogous professional roles rather than legally equivalent occupations.

The comparative scope of the present study is limited to Lithuania and the Republic of Korea; however, subsequent research may extend the analysis to additional jurisdictions to further examine different regulatory and institutional approaches to professional forest therapy. Such an expanded comparative approach could enhance the generalizability of the findings and their applicability across a broader range of legal and professional healthcare contexts.

In countries lacking specific legal regulations for forest therapy in healthcare, activities are generally governed by general service-provision rules applicable to education and training, consulting, tourism, and related sectors. At the same time, practitioners rely on ethical codes, methodologies, or standards established by the organizations that provide their training.

Although scientific literature widely associates forest therapy with health promotion, disease prevention, and the alleviation of health-related conditions, there is no unified legal definition of professional forest therapy within healthcare systems, and national approaches to its legal integration remain diverse, potentially affecting the integration of forest therapy into health systems.

The legal differences may have implications for legal uncertainty about forest therapy as part of the healthcare system and for specialists’ employment opportunities across countries because professional training varies in alignment (including qualifications, competencies, and the scope, content, duration, and quality of theoretical and practical knowledge acquired).

The analyzed training pathways differed substantially in their professional and legal status. Completion of a training program indicates that the participant has completed the requirements established by the training provider, but does not in itself constitute authorization to practice. Some programs lead to private certificates issued by training providers or professional organizations, whereas others are linked to legally recognized professional qualifications. Where applicable, professional certification represents a further form of recognition of professional competence and should not be equated with a private training certificate. Licensing or statutory authorization to practice constitutes a separate legal status, which may require completion of recognized training and satisfaction of additional regulatory requirements. Accordingly, training completion, private certification, professional certification, professional qualification, and licensing or statutory authorization to practice are treated as distinct categories in this analysis.

The findings concerning cross-border professional mobility should be interpreted with caution. Although the EU and Lithuanian legal framework contains provisions relevant to the temporary and occasional provision of services, determining their specific application to forest therapy requires a more detailed assessment of the legal status of the activity under the EU professional-qualification framework. In particular, further research is needed to examine the distinction between regulated and non-regulated professional activities, the conditions applicable in the host Member State, and the extent to which qualification, licensing, registration, or other regulatory requirements may constitute conditions for lawful professional practice and market access. Accordingly, the present study does not draw a general conclusion that the existing regulatory framework either restricts or facilitates the right to work or freedom of movement in the field of forest therapy.

The findings should not be interpreted as demonstrating that regulatory formalization necessarily improves the quality or safety of forest therapy services. The present study examined the legal and institutional framework rather than practitioner competence, patient outcomes, adverse events, patient satisfaction, or compliance with regulatory requirements. Accordingly, patient and service-user protection is considered a potential implication of regulatory formalization rather than an empirically demonstrated outcome. Further research is needed to examine how specific mechanisms, including professional accountability, scope of practice, supervision, complaints procedures, and other patient-protection measures, operate in practice.

The legal regulation may contribute to greater standardization, clearer professional responsibilities, and accountability; however, these potential benefits should be considered alongside possible barriers to professional entry and reduced flexibility in service provision. The present study does not establish a direct relationship between the level of regulatory formalization and the quality or outcomes of forest therapy services.

## 5. Conclusions

The study results revealed that the integration of professional forest therapy into national legal systems occurs in different and often fragmented ways, reflecting diverse regulatory models. The legal differences may have implications for the development of more consistent approaches to forest therapy regulation, particularly with regard to minimum professional and service standards, the reliability of the method, and the protection of service users’ rights.

The findings also suggest that regulatory approaches to forest therapy may need to be proportionate to the nature and potential risks of the services provided. A uniform requirement for full healthcare licensure may strengthen professional accountability, patient protection, and mechanisms for redress when forest therapy is provided within a clinical context. At the same time, such an approach may impose barriers to professional entry, limit flexibility and innovation, or be less appropriate for practitioners whose activities are primarily educational, preventive, or health-promotional rather than clinical. Conversely, voluntary certification may support professional diversity and accessibility, but it may also result in variability in competencies, uncertainty for service users, and less clearly defined mechanisms for complaints or professional accountability.

One possible approach, therefore, may be a tiered regulatory framework that distinguishes between general forest bathing and nature-based wellbeing activities, preventive and health promotion interventions, and clinically oriented forest therapy provided to individuals with diagnosed health conditions. Such an approach could allow regulatory requirements to be proportionate to the level of professional responsibility and potential risk associated with each type of activity. However, this remains a conceptual possibility rather than an empirically established regulatory model, and further research is needed to assess its feasibility, effectiveness, acceptability, and potential implications for both practitioners and service users.

Based on the findings of this study, a proportionate and risk-sensitive approach to the legal regulation of professional forest therapy may be considered. Rather than introducing a uniform regulatory model, policymakers and relevant professional bodies could consider establishing minimum competency requirements, clearly defined scopes of professional practice, safety and ethical standards, transparent requirements for practitioner qualifications, and appropriate referral procedures for activities involving healthcare services. Training organizations and professional associations could contribute to the development and periodic review of such standards. However, these proposals should be regarded as potential policy directions rather than empirically established solutions, and further comparative research involving additional jurisdictions and empirical evidence on patient and service-user outcomes is needed before broader regulatory recommendations can be made.

## Figures and Tables

**Table 1:** Overview of the variety of terms used to describe forest therapy-related specialists across selected countries and regions (source: compiled by the authors and based on data published on the official websites of the organizations [33,34,35,36,37,38,39] and refers to 1 January 2026).

Country/Region/ Worldwide	Nature-Based Therapy Training Provider/Responsible Organization	Certification by the Training Provider	Training and Qualification Status
Australia/Worldwide	International Nature and Forest Therapy Alliance (INFTA)	Forest Therapy Guide	Private Certification
Europe/ Worldwide	Forest Therapy Hub (FT Hub)	Nature Connection Specialist	Private Certification
		Forest Bathing Guide	Private Certification
		Forest Therapy Practitioner	Private Certification
Lithuania	Lithuanian University of Health Sciences (LSMU)	Complementary and Alternative Healthcare Forest Therapy Specialist	State-regulated Professional Qualification or (and) Licensing
North America/Worldwide	Association of Nature and Forest Therapy Guides and Programs (ANFT)	Forest Therapy Guide	Private Certification
	Nature and Systems Institute (NSI) with ANFT	Nature as Medicine Practitioner	Private Certification
Republic of Korea	Korea Forest Welfare Institute under the Korea Forest Service (KFWI) (as responsible institution for training)	Forest Therapy Instructor Level 1	State-regulated Professional Qualification or (and) Licensing
		Forest Therapy Instructor Level 2	State-regulated Professional Qualification or (and) Licensing
Sweden	Scandinavian Nature and Forest Therapy Institute (SNFTI)	Forest Bathing Guide	Private Certification
		Certified Forest Therapy Guide	Private Certification
		Eco Forest Therapy Guide	Private Certification

**Table 2:** Summary of the results of comparative analysis on key normative requirements for CAHC forest therapy training under Lithuanian 2025–2026 legal regulation (source: compiled by the authors; data refer to 1 January 2026).

Requirements for Forest Therapy Training	Applicable Until 31 December 2025 (Order No. V-557 2021, Para. 12)	Applicable from 1 January 2026 (Order No. V-1157 2025, Para. 15.3)
Total Duration/Scope	Minimum 480 h, including Theoretical Training (30–40%), Practical Sessions (40–60%), and Professional Practice (20–30%)	Minimum 60 Credits (ECTS)
Program Content Distribution	Theoretical Training (30–40%) Practical Sessions (40–60%) Professional Practice (20–30%)	Theoretical Training (45–50%) Practical Sessions (30–35%) Professional Practice (20%)
Program Distribution over Time	Not specified	Maximum 45 Credits (ECTS) taught per year

**Table 3:** Classification of forest welfare experts under the legal framework in the Republic of Korea. (Source: compiled by the authors; data refer to 1 January 2026).

Forest Welfare Expert	Category	Legal Basis
Forest Interpreter	A person who provides citizens with interpretation, guidance, and education services so that citizens can obtain knowledge about forests and develop a proper sense of value through their activities for forestry culture and recreation	Art. 2, para. 2(a) Forest Education Promotion Act 2021
Forest Guide	A person who guides and educates little children that they can cultivate their emotions and grow up to be well-rounded persons	Art. 2, para. 2(b) Forest Education Promotion Act 2021
Forest Trekking Guide	A person who provides citizens with interpretation, guidance, and education services so that they can enjoy mountaineering or trekking (referring to learning about the history and culture of an area, enjoying the scenery thereof, and improving health by walking along the trail) safely and comfortably	Art. 2, para. 2(c) Forest Education Promotion Act 2021
Forest Therapy Instructor	A person qualified to instruct forest healing as a means of immune-strengthening and health-promoting activities, which utilize a variety of elements of the forest, including scents and scenic views	Art. 11-2 Forestry Culture and Recreation Act 2023
Instructor of Forest Leisure Sports	A person qualified to instruct leisure sports in a forest to promote forest healing through leisure sports in a forest, which means leisure sports conducted as adventure or as experience in a forest	Art. 12 Forestry Culture and Recreation Act 2023

## Data Availability

The original contributions presented in this study are included in this article. The sources of publicly available data are clearly identified in this manuscript. The number of individuals who completed the forest therapy training program is reported in this article and may be verified through the relevant institutional sources, including the LSMU Postgraduate Studies Centre, which maintains the relevant administrative records. Further inquiries can be directed to the corresponding author.

## Abbreviations

The following abbreviations are used in this manuscript:

ANFT	Association of Nature and Forest Therapy Guides and Programs
CAHC	Complementary and Alternative Health Care
FCES	Forest Cultural Ecosystem Services
FT Hub	Forest Therapy Hub
INFTA	International Nature and Forest Therapy Alliance
ISFT	International Society of Forest Therapy
ISO	International Organization for Standardization
IUCN	International Union for Conservation of Nature
LSMU	Lithuanian University of Health Sciences
TCIM	Traditional, Complementary and Integrative Medicine
WHO	World Health Organization
